# STITCH links cellular morphology and gene expression in spatial transcriptomics

**DOI:** 10.64898/2026.06.07.730714

**Published:** 2026-06-11

**Authors:** Shashwat Kumar, Yingxiao Shi, Tuulia Vallius, Chi-Ping Day, P.-A. Absil, Anuj Srivastava, Sridhar Hannenhalli, Vishaka Gopalan

## Abstract

In situ spatial (ISS) sequencing can uncover co-variation between cellular morphology and gene expression in vivo. However, a principled and interpretable mathematical representation of morphology has not yet been applied in this context. In particular, current deep learning-based representations of cell images confound a cell’s shape with its size. We present an interpretable representation of cellular boundary contours, based on tangent principal component analysis (TPCA) in a Kendall shape manifold, that captures size-independent contour shape features. This approach successfully recovers shape-perturbing genes in an RNAi screen than a previous metric geometry-based approach. We build on TPCA to develop STITCH (Shape–TranscriptomIc Correlation and Harmonization), an approach to reveal covariation between cell morphology with gene expression in ISS datasets. In a Xenium dataset, STITCH outperforms a deep learning-based approach in both recovering the layered organization of keratinocytes and a spatial gradient in nuclear eccentricity. Across samples in a melanoma CosMx dataset, STITCH reproducibly associates elongated and triangular fibroblasts with proximity to malignant cells and myofibroblast-like transcriptional program. Finally, STITCH independently recovers a known link between mesenchymal-like malignant cell states and increased cell area in two melanoma cohorts. STITCH can thus yield interpretable morphology–transcriptome relationships across cell types, patients, and spatial transcriptomics platforms.

## Full Text Availability

The license terms selected by the author(s) for this preprint version do not permit archiving in PMC. The full text is available from the preprint server.

