# Supplementary material for: KSTITCH links cellular morphology and gene expression in spatial transcriptomics
Source: bioRxiv. 2026 Aug 26:2026.06.07.730714. Originally published 2026 Jun 11. Preprint. [Version 2] doi: 10.64898/2026.06.07.730714 (PMC13278165; doi:10.64898/2026.06.07.730714)
Supplement: Supplementary file 1 [file NIHPP2026.06.07.730714v2-supplement-4.pdf]

(which was not certified by peer review) is the author/funder. This article is a US Government work. It is not subject to copyright under 17 U
105 and is also made available for use under a CC0 license.

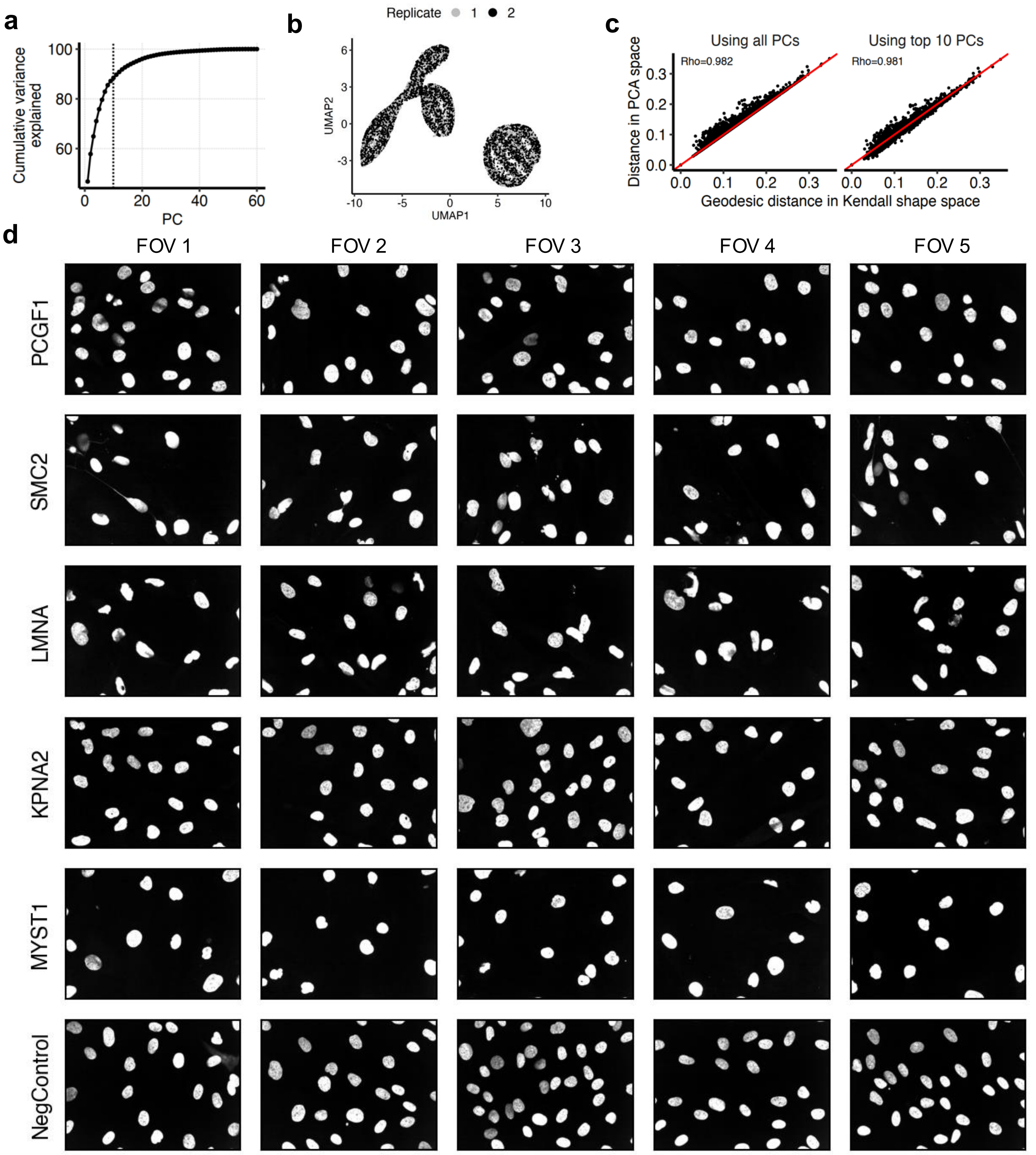

Supp. Figure 1 : (a) Variance in Schibler. et al data (y-axis) cumulatively explained by considering successive principal components (x-axis). Vertical line drawn at x = 10 represents the number of PCs used in analyses. (b) UMAP embedding of 20,000 sampled nuclei (~1% of dataset), with each point representing a nucleus and colored based on the replicate in which it was imaged. UMAP coordinates were computed based on the first ten shape PCs. (c) Scatter plot of geodesic distances (x-axis) and distances in TPCA space (y-axis) between pairs of nuclei sampled (N = 1000) from Schibler et. al dataset. Distances are calculated based on either all PCs (left panel) or top ten PCs (right panel. (d) Contrast-enhanced images of nuclei (DAPI stains) after knockdown of genes labelled as shape genes in Schibler et. al (PCGF1, SMC2, LMNA) and knockdowns of genes detected by our TPCA approach as shape genes (KPNA2,MYST1) but not detected by Schibler et. al. Nuclei from negative controls (NegControl) are along the bottom row . Each column shows nuclei from different fields of view associated with each perturbation.

(which was not certified by peer review) is the author/funder. This article is a US Government work. It is not subject to copyright under 17 U
105 and is also made available for use under a CC0 license.

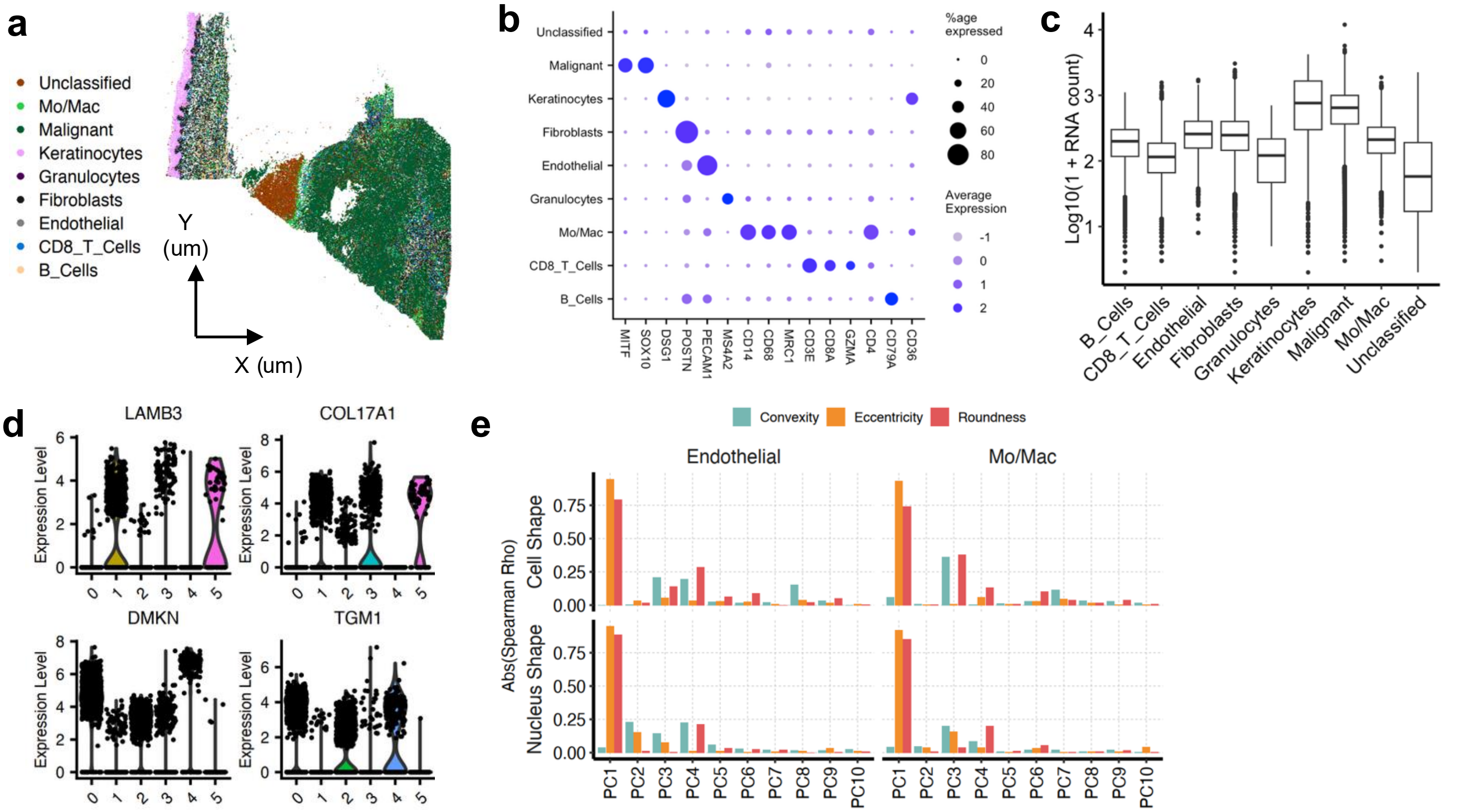

Supp. Figure 2 :

(a) Spatial distribution of annotated cell types in Xenium Prime dermal melanoma dataset.

(b) Marker genes used to determine identities of all cell types in Xenium Prime dermal melanoma dataset.

(c) Distributions of RNA counts in each cluster.

(d) Genes used to label keratinocyte subtypes in dataset.

(e) Absolute correlation coefficient (Spearman Rho) between cellular shape PCs and convexity, eccentricity and roundness (top row) of endothelial cells and monocytes/macrophages. Correlation coefficients of nuclear shape PCs with the same shape descriptors are shown in the bottom row.

(which was not certified by peer review) is the author/funder. This article is a US Government work. It is not subject to copyright under 17 U
105 and is also made available for use under a CC0 license.

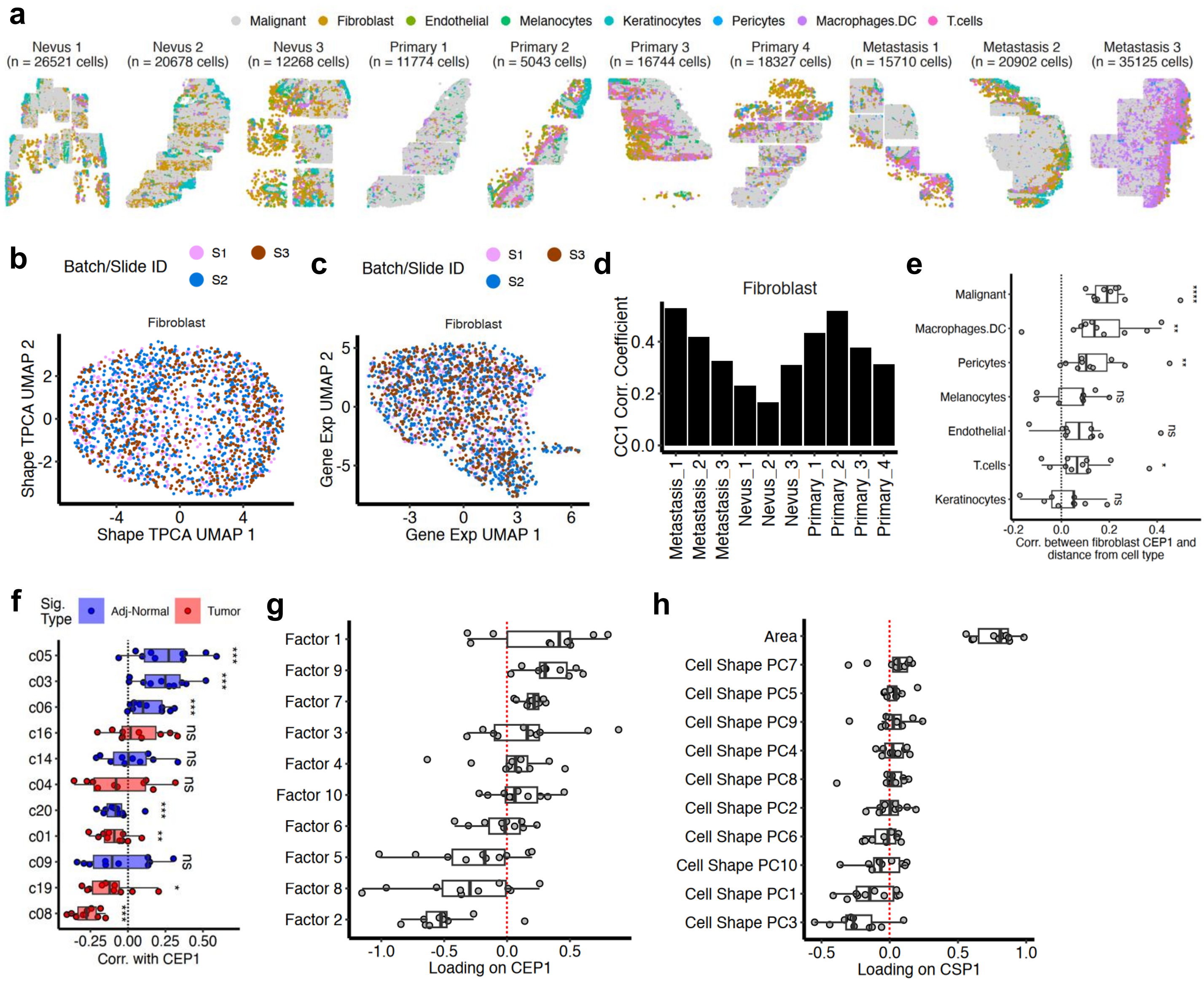

Supp. Figure 3 : (a) Spatial coordinate plot of all CosMx samples Love et. al melanoma cohort, with color indicating cell type. (b) UMAP coordinates of fibroblasts across all samples based on their cellular shape TPCA embeddings and colored by the CosMx slide from which they originate. (c) UMAP coordinates computed from NMF embeddings of fibroblasts pooled across samples. (d) Correlation coefficient associated with the first canonical correlation (between CSP1 and CEP1) when STITCH is run separately on fibroblasts from each sample of Love et. al cohort. Bars in black indicate significant ($p < 0.05$) correlations in that sample. (e) Correlation of fibroblast CEP1 score from each sample with distance from each cell type. Each dot represents a different sample. (f) Correlation between fibroblast CEP1 of each sample and expression of fibroblast signatures from Gao et. al. Each dot represents correlations from a single sample. (g) Loadings of each shape PC and cellular area on CSP1 from fibroblasts of each sample. Each dot represents loadings from a single sample. (h) Loadings of each factor on CEP1 from fibroblasts of each sample. Each dot represents loadings from a single sample.

(which was not certified by peer review) is the author/funder. This article is a US Government work. It is not subject to copyright under 17 U
105 and is also made available for use under a CC0 license.

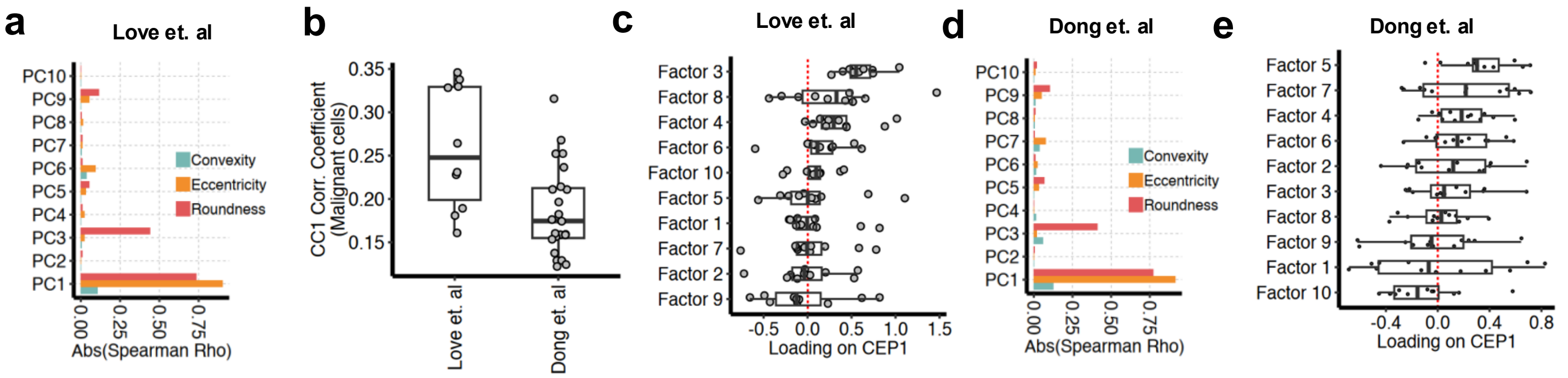

Supp. Figure 4 (a) Correlation of malignant cell boundary PC shape modes from Love et. al cohort with cell eccentricity, roundness and convexity. (b) Distribution of CC1 correlation coefficients obtained from running STITCH on malignant cells from samples Love et. al cohort (n=10) and Dong et. al cohort (n=25). (c) Loadings of gene expression factors derived from malignant cells in Love et. al cohort on CEP1. Each dot represents a loading in a different sample from Love et. al cohort. (d) Correlation of malignant cell boundary PC shape modes from Dong et. al cohort with cell eccentricity, roundness and convexity. (e) Loadings of gene expression factors derived from malignant cells in Dong et. al cohort on CEP1. Each dot represents a loading in a different sample from Dong et. al cohort.
